# Supplementary material for: Inflammatory factors and risk of lung adenocarcinoma: a Mendelian randomization study mediated by blood metabolites
Source: Front Endocrinol (Lausanne). 2024 Aug 27;15:1446863. doi: 10.3389/fendo.2024.1446863 (PMC11384989; doi:10.3389/fendo.2024.1446863)
Supplement: Supplementary file 1 [file DataSheet1.zip › supplementary files/S2/GCST90200937/forest.pdf]

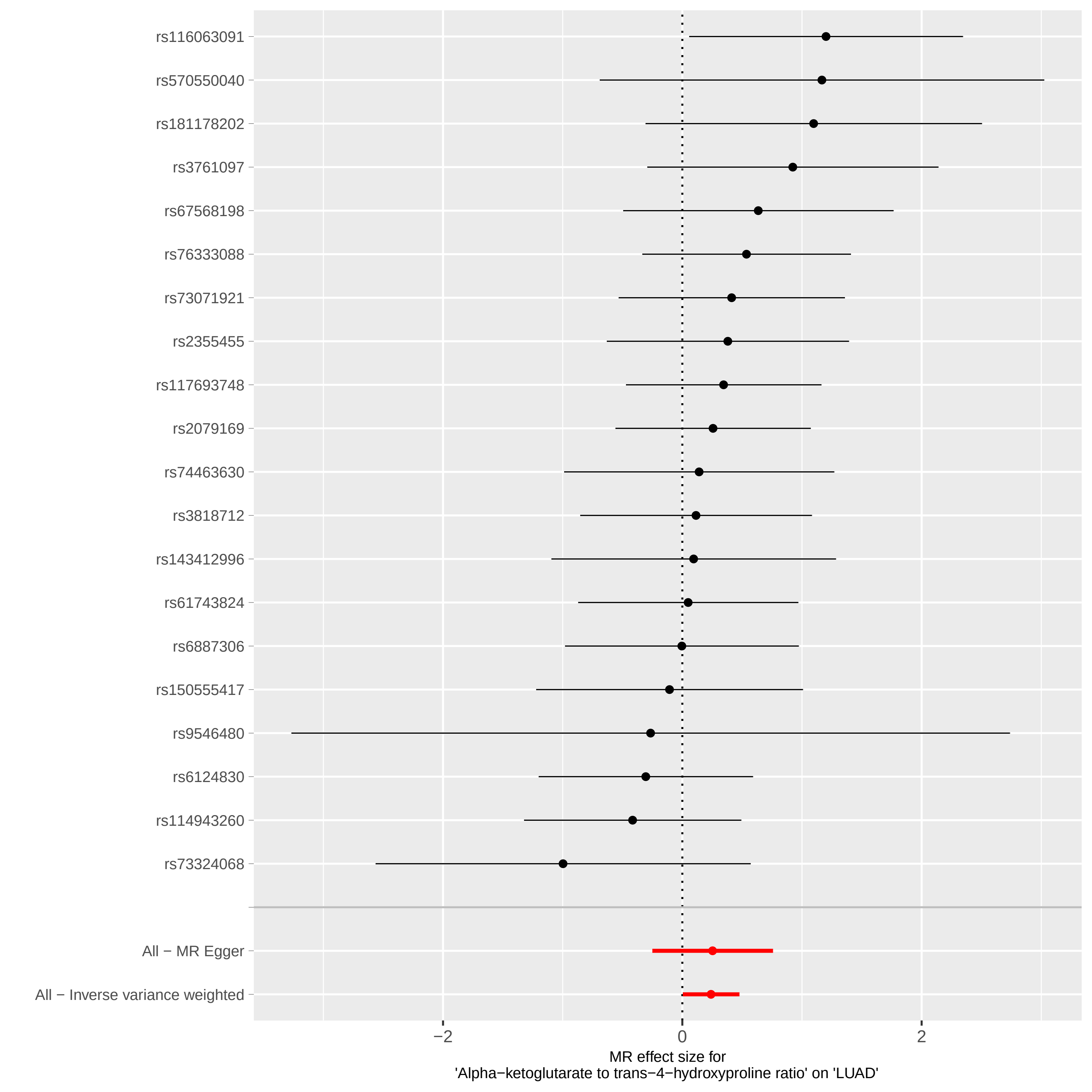

rs116063091
rs570550040
rs181178202
rs3761097
rs67568198
rs76333088
rs73071921
rs2355455
rs117693748
rs2079169
rs74463630
rs3818712
rs143412996
rs61743824
rs6887306
rs150555417
rs9546480
rs6124830
rs114943260
rs73324068
All − MR Egger
All − Inverse variance weighted
−2
0
2
MR effect size for
'Alpha−ketoglutarate to trans−4−hydroxyproline ratio' on 'LUAD'
